# Guanidinium 5,5′‐Azotetrazolate: A Colorful Chameleon for Halogen‐Free Smoke Signals

**DOI:** 10.1002/anie.202007489

**Published:** 2020-06-25

**Authors:** Teresa Küblböck, Gaspard Angé, Greta Bikelytė, Jiřina Pokorná, Radovan Skácel, Thomas M. Klapötke

**Affiliations:** ^1^ Ludwig-Maximilian University of Munich Butenandtstrasse 9 81377 Munich Germany; ^2^ ENSTA Bretagne 2, rue François Verny 29806 Brest Cedex 09 France; ^3^ EXLOSIA a.s. Semtín 107, PSČ 530 02 Pardubice Czech Republic

**Keywords:** azo compounds, halogen-free, pyrotechnics, smoke, sustainable chemistry

## Abstract

A progressive halogen‐free multicolored smoke system to obtain white, red, violet, yellow, green, and blue smoke color is presented. The nitrogen‐rich salt guanidinium 5,5′‐azotetrazolate (GZT), which is usually applied as a gas generator or propellant ingredient, was combined with different smoke dyes (Solvent Red 1, Solvent Violet 47, Solvent Green 3, Solvent Yellow 33). These two‐component smoke mixtures offer a convenient and safe multicolor approach without the need for potassium chlorate or any other hazardous material. The common smoke characteristics with respect to burn time/burn rate, yield factor, transfer rate, as well as energetic properties were determined and compared with classic chlorate‐based formulations currently used. To the best of our knowledge, nothing comparable is known in the literature and a completely new research area in modern pyrotechnics is opened.

## Graphical abstract

**Nitrogen‐rich smoke**: The first halogen‐free multicolored smoke system has been designed. In this approach, guanidinium 5,5′‐azotetrazolate (GZT) was simply mixed with various dyes to obtain the desired color impressions. The absence of controversial potassium chlorate results in a much safer method for smoke generation.

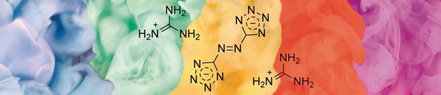

The application of colored smoke as daylight fireworks as well as for color effects during photography shootings or fashion shows has become very popular in the last few years.^1^ Colored smoke is now known and accessible to a much broader target group than before, and it is more important than ever to ensure safe handling and reduce health concerns during their use by untrained people. Moreover, there is an increasing priority regarding issues such as sustainability and environmental effects.^2^ However, to date research efforts have only been driven from a military point of view. In this context, they are commonly applied as reliable non‐electronic communication tools for either ground‐to‐ground or ground‐to‐air signaling.^3^ In 2017, the Strategic Environmental Research and Development Program (SERDP) reported the need for so‐called “next generation pyrotechnics” that reduce the environmental and health effects.^2^ The field of smoke‐producing pyrotechnics offers a wide variety of research possibilities.

The first colored smoke signals consisted mainly of an organic dye, sulfur, potassium chlorate, sodium bicarbonate, and optional amounts of kerosene or tricalcium phosphate.^4^ Sulfur in combination with potassium chlorate offers a low ignition and combustion temperature; therefore, it is the perfect candidate for low‐temperature smoke.3a, 5 The color impression can easily be obtained by a sublimation‐recondensation mechanism.3a, 5, 6 In detail, the pyrotechnic mixture provides the energy to sublime the dye, which can subsequently recondense as small particles.3a, 6b, 6c Maintaining the lower combustion temperatures is mandatory, otherwise the organic dye would be burned rather than sublimed.6b, 7 Finally, the gaseous combustion products disperse the emerging dye particles, which results in a dense colored smoke cloud.6c A big step forward was the substitution of sulfur by alternative fuels.^5^, ^8^ During the combustion of sulfur‐based smoke mixtures, hazardous SO_2_ is formed that causes a burning sensation in the lungs when inhaled.^5^ Fortunately, sugar‐based fuels such as sucrose or lactose act in a comparable manner to sulfur when paired with potassium chlorate.^5^, ^9^ Sugar is considered a less toxic alternative, since the resulting combustion products are limited to mainly harmless water and carbon dioxide.^5^, ^10^

Another big challenge in today's research is the elimination of halogens and halogen‐containing molecules.^2^, ^5^ In the case of smoke signals, potassium chlorate still seems to be the only suitable oxidizing agent to ensure the optimal temperature range for dye sublimation.6b, 11 Several serious issues arise from the use of potassium chlorate: It is highly reactive and tends to undergo spontaneous ignition, particularly in combination with combustible low‐melting fuels.6b, 11 Furthermore, as a consequence of its water solubility and persistence, it can cause problems to aquatic life as it is toxic.^12^ The combustion products, in particular, are an underestimated risk in pyrotechnics. Chlorates in combination with organic materials are known to form toxic and carcinogenic chlorinated organic compounds such as polychlorinated dibenzo‐*p*‐dioxins (PCDD) or dibenzofurans (PCDF). These gaseous side products can be inhaled very easily during combustion.^13^ Nevertheless, because of the lack of sufficient alternatives, potassium chlorate is still commonly used as the oxidizer in smoke‐producing mixtures.

In 2015, the very first chlorine‐free red flare was investigated by Sabatini et al.^14^ Their strategy was to add molecules such as hexamine or 5‐amino‐1*H*‐tetrazole instead of unwanted potassium perchlorate to a common flare formulation. They concluded that these nitrogen‐rich fuels have a deoxidizing effect on the flame and thus result in a lower combustion temperature.^14^ This concept was successfully transferred to smoke‐generating pyrotechnics by Glück et al. for both colored and white smoke formulations.^15^ It was possible to replace sugar by hexamine and 5‐amino‐1*H*‐tetrazole as the main fuel, which led to an overall more persistent and thick smoke cloud.^15^, ^16^ Another positive side effect is the release of a high volume of gaseous products such as N_2_, which further disperse the dye particles.^17^ Nevertheless, until now it was not possible to eliminate potassium chlorate in smoke, not even through the use of nitrogen‐rich molecules.3a, 3b, 4b, 5, 6b, 15, 16

The next logical step is to find a suitable nitrogen‐rich molecule for smoke signals, which—in the best case—can be applied without any potassium chlorate or other halogen sources. An extensive literature research revealed an interesting molecule with an overall nitrogen content of 78.9 %: guanidinium 5,5′‐azotetrazolate (GZT, Figure 1).^18^ GZT is a bright yellow powder and burns with white smoke.17a, 19 As a consequence of its beneficial properties—ranging from high thermal stability, insensitivity towards external stimuli, to desirable low combustion temperatures—it is expected to be an ideal candidate for application in clean propulsion systems, new gas‐generators, and low signature propellants.17a, 17c, 18, 19, 20 GZT derives its power from a high heat of formation in combination with the release of a large gas volume, which is mainly cool and inert because of the formation of N_2_.17a, 17c, 18, 19, 20b As a consequence of its beneficial properties, GZT was also considered as a gas‐generating compound in smoke signals.

**Figure 1:**
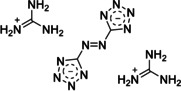
Guanidinium 5,5′‐azotetrazolate (GZT).

First, the energetic properties of GZT were reviewed (Table 1, see also Table S9 in the Supporting Information). GZT burned with the generation of moderately strong white smoke and a burn time of 19 s, which correlated with the burn rate (11.8 mm s^−1^). The impact sensitivity was confirmed to be 35 J, and it was not sensitive towards friction and electrostatic discharge.17a GZT decomposed at 239 °C. The nonsensitive energetic properties in combination with the high decomposition temperature are advantageous for modern pyrotechnics, since they guarantee safe preparation, storage, and handling.

**Table 1:** Selected properties as well as sensitivities of GZT and GZT‐based smoke in comparison with chlorate‐based references.15b The exact composition of formulations, the theoretical background, as well as experimental setup is described in the Supporting Information.^[a]^

	BT [s]	BR [mm s^−1^]	*m_a_* [mg]	*Y* [%]	*m_d_* [mg]	*T%* [%]	*T*_dec_ [°C]
GZT	19	11.8	654	32	–	–	239
W3	26	6.9	582	29	–	–	215
Y3	26	5.3	783	38	164	55	239
R3	25	5.6	745	36	169	56	239
V3	29	5.6	779	38	–	–	237
B3	24	6.8	726	35	–	–	239
G3	19	7.3	715	35	–	–	238
Ref‐W	38	–	693	35	–	–	203
Ref‐Y	13	–	670	33	435	73	178
Ref‐R	21	–	729	36	514	86	172
Ref‐V	15	–	652	32	–	–	178
Ref‐G	19	–	642	32	–	–	172

[a] BT=burn time (2.0 g scale); BR=linear burn rate (5.0–6.0 g scale); *m_a_*=mass of produced aerosol (2.0 g scale); Y=yield factor; *m_d_*=dye content present in produced aerosol; *T%*=transfer rate; *T*_dec_=temperature of decomposition.

The newly developed smoke formulations should combine acceptable smoke performance, an economical manufacturing process, as well as safe application (also by untrained people).^2^ For this reason, the smoke system must be as simple and efficient as possible. As a starting point, GZT was quick‐mixed with various proportions of dye (5–35 %) to develop two‐component smoke. The combustion of GZT was assumed to provide enough heat for dye sublimation, and the white smoke generated should switch its color impression. All GZT‐based smoke mixtures were characterized according to their burning behavior (burn time: *BT*, burn rate: *BR*), smoke quality (mass of aerosol *m_a_*, yield factor *Y*, mass of dye present in the aerosol *m_d_*, transfer rate *T%*) as well as energetic properties and then further compared with classic chlorate/sugar‐based smokes. The reference formulations consisted of different dyes (terephthalic acid, Disperse Red 9, Violet Dye Mix, Solvent Yellow 33, Solvent Green 3), potassium chlorate, sucrose or hexamine, and magnesium carbonate hydroxide pentahydrate (see Table S2 and Table S8).

It is obvious to expect GZT to be a white smoke generator (Figure 2 a). For this reason, we determined the mass of aerosol formed during combustion (654 mg) as well as the yield factor (32 %). These results are very similar to the white hexamine/chlorate reference (693 mg, 35 %). GZT has the advantage of its superior impact sensitivity (35 J instead of 10 J); however, for application, the spectral properties must also be considered in detail.

**Figure 2:**
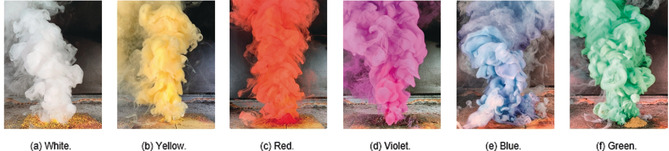
Burning of GZT‐based colored smoke formulations.

In common less‐toxic white smoke signals the dye of choice is terephthalic acid (TA), since it is easily accessible at moderate prices and has acceptable properties.3a, 5, 21 To improve the performance of GZT alone, terephthalic acid was added stepwise (see Tables S1 and S2). We found that the upper limit is 15 % TA content; above that, it was no longer possible to ignite the GZT/TA mixture. The sublimation temperature of TA is 402 °C and, therefore, slightly higher than anthraquinone or quinoline dyes (approximately 300–350 °C).^12^ As a result, the sublimation of TA might consume too much energy from the system, since smoke properties dropped significantly to 582 mg aerosol and 29 % yield. For this reason, the combination of GZT and TA was excluded from further investigation.

Environmentally benign multicolored smoke signals are a current research topic, because the variety of application areas as well as the demand is rising.^1^, ^2^, ^3^ Therefore, GZT was simply mixed with various dyes to obtain red (Solvent Red 1), violet (Solvent Violet 47), yellow (Solvent Yellow 33), blue (Solvent Green 3), and green (Solvent Yellow 33 + Solvent Green 3) color impressions. The same procedure as for white smoke was applied, whereby the dyes were added stepwise to GZT (see Tables S3–S7). Since GZT burns with white smoke, it is advantageous to have as much dye content as possible, otherwise, the color impression might be lacking. As observed for TA, the upper limit for colored dyes was also 15 % for constant igniting and burning behavior. Initial tests with these two‐component dye/GZT mixtures showed the generation of strong and thick smoke (Figure 2). The expected color impression is slightly lighter because of the presence of GZT; however, it is still clearly recognizable. The new smoke system was set to 15 % dye in combination with 85 % GZT for all colors. It is noteworthy that the dye content of the GZT system is half as much as for reference formulations, which needs to be considered during characterization and comparison. In contrast to the simple two‐component GZT system, the references were based on sucrose and potassium chlorate (see Table S8).

The burn times of colored smokes were in the range of 24–29 s, with **G3** as the only exception (19 s). In contrast, the underlying redox reaction of sucrose/chlorate mixtures is much more violent and results in faster smoke generation and shorter burn times (13–21 s). The yield factor of pure GZT was 32 %, which could be further increased by the addition of dye (35–38 %). The yield factor as well as the mass of aerosol *m_a_* is in a similar range as the reference formulations. These results indicated that the dye particles have been dispersed by GZT. To determine whether the organic dyes were transformed or destroyed during combustion,^3^ HPLC analysis was performed to quantify the exact amount of dye present in the aerosol.15b, 15c The resulting transfer rate *T%* is a measure of the effectiveness of smoke mixtures in dispersing the dye rather than combusting it. In comparison to red and yellow reference formulations (73–86 %), the two‐component GZT system reached a transfer rate of up to 55–56 %. This means, that about 55 % of the dye particles in the pellet are sublimed and present in the generated smoke cloud. It should be noted that only half of the dye content was used in the GZT system than in the reference mixtures (15 % instead of 30 %). Therefore, we suggest that the probability of sublimation might be influenced by the significantly different particle content. Nonetheless, these novel and simple nitrogen‐rich smokes already showed a promising trend to reach similar smoke performance as in the currently used formulations.

A special highlight is the overall nonsensitive energetic properties of GZT in combination with a high decomposition temperature (239 °C). The addition of dye resulted in formulations that are not sensitive to impact, friction, or electrostatic discharge. The decomposition temperatures of all the colored smoke mixtures were in the same range as that of GZT. In comparison, the use of the sugar/chlorate mixture is very questionable, since it is known to undergo spontaneous reactions as well as being unpredictable.3a, 6b, 11b, 22 This creates high risks during production and storage.6b The elimination of the controversial potassium chlorate results in a much safer way of generating smoke. This is also advantageous for untrained people, since they can use pyrotechnics without exposing themselves to the danger of unexpected reactions.

As potassium chlorate and sugar are essential materials in the global industry, they are available in adequate quantities at reasonable prices. In contrast, the nitrogen‐rich salt GZT is more complex to produce and, therefore, more expensive. However, common in‐use smokes consist not only of oxidizer and fuel, but a coolant and other additives are also required. It should be considered that GZT can be applied as a single compound, which greatly simplifies the manufacturing process, purchasing, and storage as well as the previously discussed safety issues.

Herein we have discussed halogen‐free two‐component smoke mixtures based on the nitrogen‐rich salt guanidinium 5,5′‐azotetrazolate, which was combined with various dyes. The examined system consisted of 15 % dye and 85 % GZT. First, GZT was evaluated in terms of its energetic properties and its applicability to generate white smoke. The colored system was based on using various organic dyes: Solvent Red 1, Solvent Violet 47, Solvent Yellow 33 as well as Solvent Green 3. These simple two‐component smoke systems were able to generate thick colored smoke clouds without the need for any potassium chlorate. A similar yield factor and mass of aerosol was observed as for commonly used sucrose/chlorate mixtures. Only the transfer rates were slightly lower than for the references.

Guanidinium 5,5′‐azotetrazolate is only one representative of the group of nitrogen‐rich gas‐generating compounds. There might be other possible candidates, which could further improve the effectiveness of smoke mixtures. For these future compounds, the dye content should be increased to ensure unique and brilliant color impressions. One of the most challenging tasks in pyrotechnics is the toxicity of combustion products. Potassium chlorate in combination with organic material leads to carcinogenic polychlorinated compounds, which can now be prevented by using nitrogen‐rich smokes. However, the toxicity of these combustion products should also be further evaluated to satisfy health aspects.

## Conflict of interest

The authors declare no conflict of interest.

## Supporting information

As a service to our authors and readers, this journal provides supporting information supplied by the authors. Such materials are peer reviewed and may be re‐organized for online delivery, but are not copy‐edited or typeset. Technical support issues arising from supporting information (other than missing files) should be addressed to the authors.

Supplementary
