# Supplementary material for: Guanidinium 5,5′‐Azotetrazolate: A Colorful Chameleon for Halogen‐Free Smoke Signals
Source: Angew Chem Int Ed Engl. 2020 Jun 25;59(30):12326–30. doi: 10.1002/anie.202007489 (PMC7383485; doi:10.1002/anie.202007489)
Supplement: Supplementary file 1 — Supplementary [file ANIE-59-12326-s001.pdf]

Angewandte Chemie
Eine Zeitschrift der Gesellschaft Deutscher Chemiker

Supporting Information

Guanidinium 5,5′-Azotetrazolate: A Colorful Chameleon for Halogen-Free Smoke Signals

*Teresa Küblböck, Gaspard Angé, Greta Bikelytė, Jiřina Pokorná, Radovan Skácel, and Thomas M. Klapötke**

anie_202007489_sm_miscellaneous_information.pdf

Table of Contents

Experimental Procedures p.1
Results and Discussion p.2
References p.5

Experimental Procedures

CAUTION! The described pyrotechnical mixtures might initiate during preparing, handling or manipulating! They are potential explosives, which are sensitive to environmental stimuli such as impact, friction, heat, and electrostatic discharge. Please handle these materials with care! Precautionary measures are mandatory and protective equipment like safety glasses, face shields, leather coats, Kevlar® gloves, and ear protectors is highly recommended.

Chemicals. Sucrose (≥99%), terephthalic acid (98%), Solvent Red 1 (reagent grade) and magnesium carbonate hydroxide pentahydrate (BioXtra) were purchased from Sigma-Aldrich. 5-Amino-1*H*-tetrazole (98%) was purchased from abcr chemicals. Potassium chlorate (≥99%) was purchased from Grüssing GmbH. Disperse Red 9, Solvent Green 3, Solvent Yellow 33, Solvent Violet 47 and Violet Mix Smoke Dye were purchased from Nation Ford Chemical. Guanidinium 5,5'-azotetrazolate was donated by Explosia.

Sample Preparation. All pyrotechnic samples were prepared in 2.0 g scale using the same procedure in order to ensure the reproducibility. Therefore, the different ingredients were weighed into a sample glass according to their respective weight percentages in the formulations. Each sample was transferred into a porcelain mortar and carefully ground to a homogeneous powder. After grinding, the binder solutions were added followed by a curing step. The so-prepared compositions were ground again and then, pressed into a cylindrical shape with the aid of a tooling die using a hydraulic press with a dead load of 2.0 t for 3.0 s. Each pellet was ignited using a resistance heating Kanthal® A1 wire (FeCrAl, 0.8 mm diameter, 2.9 Ω m^{-1}). For each evaluated composition, three pellets were tested, and the results were averaged.

Burn Rate. The smoke mixture (5.0−6.0 g) was pressed into a cardboard roll (2.7 mm wall thickness) with cylindrical shape (1.0 cm inner diameter, 9.0 cm height) sealed on one side. Each pellet was ignited using a resistance heating Kanthal® A1 wire (FeCrAl, 0.8 mm diameter, 2.9 Ω m^{-1}) and the whole burn was recorded with a digital video camera. The compositions were tested four times and the results were averaged. The mass burn rate BR was determined by dividing the pellet mass m_p by the burn time BT according to:

$$BR = {}^{m_p}/_{BT}\,, \qquad (1)$$

while the linear burn rate was calculated by dividing the pellet length l_p by the burn time:

$$BR = {}^{l_p}/_{BT}\,. \qquad (2)$$

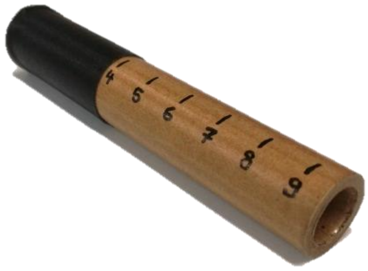

Figure S1. Cardboard roll for determination of burn rate.

Aerosol and Yield Factor.[1] The mass of produced aerosol m_a was calculated according to:

$$m_a = |\Delta m_{setup}| - |\Delta m_{bowl}| , \quad (3)$$

where Δm_{setup} is the mass difference of the whole setup before and after ignition and Δm_{bowl} is the mass difference of the bowl with pellet before and after ignition. Using the mass of produced aerosol m_a led to the yield factor Y:

$$Y = {}^{m_a}/_{m_p} \times 100 . \quad (4)$$

HPLC Strategy.[1-2] After ignition, the arising aerosol not only consists of recondensed dye, but also of other unknown combustion products. Therefore, it is necessary to investigate the collected aerosol with respect to its exact dye content by HPLC analysis. The transfer rate $T\%$ is defined as quotient between dye content present in aerosol m_d divided by the amount of dye in the pellet m_{pd}:

$$T\% = {}^{m_d}/_{m_{pd}} \times 100 . \quad (5)$$

For dye quantification a Shimadzu Prominence® HPLC with LC-20AD pump module and SPD-M20A Diode Array Detector and LabSolutions v5.86 software was used. The analytical column was a Phenomenex Kinetex® (2.6 µm Biphenyl, 100 Å, 150 × 4.6 mm).

Mobile Phase: B = water, C = acetonitrile
Gradient: 0 min (50% B, 50% C), 5 min (10% B, 90% C), 9 min (10% B, 90% C), 14 min (50% B, 50% C)
Time: 15 min
Flow rate: 0.5 mL min^{-1}
Injection volume: 1 µL
Oven temperature: 40°C
Sample temperature: 15°C

Sensitivity Data.[3] The impact and friction sensitivities were determined using a BAM Drophammer and a BAM Friction Tester. The sensitivities of the compositions are indicated according to the UN Recommendations on the Transport of Dangerous Goods (+). Impact: insensitive >40 J, less sensitive >35 J, sensitive >4 J, very sensitive <4 J; friction: insensitive >360 N, less sensitive = 360 N, sensitive 360 N > x > 80 N, very sensitive <80 N, extreme sensitive <10 N. Electrostatic discharge was measured with an OZM small-scale electrostatic spark X SPARK 10. ESD: sensitive <0.1 J, insensitive >0.1 J. The thermal stability was carried out using an OZM Research DTA 552 Ex Differential Thermal Analyzer with a heating rate of 5°C min^{-1}.

Results and Discussion

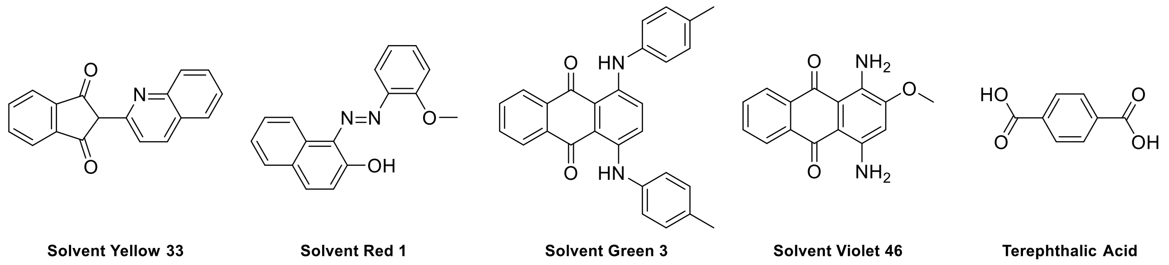

Figure S2. List of applied dyes in presented study.[4]

Table S1. White-colored smoke formulations **GZT** and **W1**–**W7**.

	**Terephthalic Acid**/wt%	**GZT**/wt%
**GZT**	0	100
**W1**	5	95
**W2**	10	90
**W3**	15	85
**W4**	20	80
**W5**	25	75
**W6**	30	70
**W7**	35	65

Table S2. White hexamine-based reference smoke formulations.[5]

	**Terephthalic Acid**/wt%	$KClO_3$/wt%	**Hexamine**/wt%	**MCHP**/wt%
**Ref-W**	45	27.5	22	5.5

Table S3. Yellow-colored smoke formulations **Y1**–**Y7**.

	**Solvent Yellow 33**/wt%	**GZT**/wt%
**Y1**	5	95
**Y2**	10	90
**Y3**	15	85
**Y4**	20	80
**Y5**	25	75
**Y6**	30	70
**Y7**	35	65

Table S4. Red-colored smoke formulations **R1**–**R7**.

	**Solvent Red 1**/wt%	**GZT**/wt%
**R1**	5	95
**R2**	10	90
**R3**	15	85
**R4**	20	80
**R5**	25	75
**R6**	30	70
**R7**	35	65

Table S5. Violet-colored smoke formulations **V1**–**V7**.

	**Solvent Violet 47**/wt%	**GZT**/wt%
**V1**	5	95
**V2**	10	90
**V3**	15	85
**V4**	20	80
**V5**	25	75
**V6**	30	70
**V7**	35	65

Table S6. Blue-colored smoke formulations **B1**–**B7**.

	**Solvent Green 3**/wt%	**GZT**/wt%
**B1**	5	95
**B2**	10	90
**B3**	15	85
**B4**	20	80
**B5**	25	75
**B6**	30	70
**B7**	35	65

Table S7. Green-colored smoke formulations **G1**–**G7**.

	**Solvent Green 3**/wt%	**Solvent Yellow 33**/wt%	**GZT**/wt%
**G1**	3.3	1.7	95
**G2**	6.7	3.3	90
**G3**	10	5	85
**G4**	13.3	6.7	80
**G5**	16.7	8.3	75
**G6**	20	10	70
**G7**	23.3	11.7	65

Table S8. Multi-colored sucrose-based reference smoke formulations fulfilling the concept of fuel mixes.[2]

	**Solvent Yellow 33**/wt%	**Solvent Green 3**/wt%	**Disperse Red 9**/wt%	**Violet Dye Mix**/wt%	$\mathbf{KClO_3}$/wt%	**Sucrose**/wt%	**MCHP**/wt%
**Ref-Y**	30	−	−	−	28	28	14
**Ref-R**	−	−	30	−	28	28	14
**Ref-V**	−	−	−	30	28	28	14
**Ref-G**	10	20	−	−	28	28	14

Table S9. Properties as well as sensitivities of GZT and GZT-based smokes in comparison with chlorate- / sucrose-based references.[2]

	**BT**/s	**BR**/mm s^{-1}	**BR**/g s^{-1}	$\mathbf{m_a}$/mg	**Y**/%	**RH**/%	$\mathbf{m_d}$/mg	**T%**/%	**IS**/J	**FS**/N	**ESD**/J	$\mathbf{T_{dec}}$/°C
**GZT**	19	11.8	0.07	654	32	58	−	−	35	360	1.0	239.2
**W3**	26	6.9	0.04	582	29	61	−	−	40	360	1.0	214.9
**Y3**	26	5.3	0.03	783	38	63	164	55	40	360	1.0	238.5
**R3**	25	5.6	0.03	745	36	58	169	56	40	360	1.0	238.8
**V3**	29	5.6	0.04	779	38	58	−	−	40	360	1.0	236.5
**B3**	24	6.8	0.04	726	35	62	−	−	40	360	1.0	239.1
**G3**	19	7.3	0.04	715	35	61	−	−	40	360	1.0	237.6
**Ref-W**	38	−	0.26	693	35	31	−	−	10	360	0.6	203
**Ref-Y**	13	−	0.43	670	33	29	435	73	40	360	0.3	178
**Ref-R**	21	−	0.41	729	36	28	514	86	40	360	0.6	172
**Ref-V**	15	−	0.29	652	32	28	−	−	30	360	0.7	178
**Ref-G**	19	−	0.43	642	32	28	−	−	30	360	0.2	172

BT = burn time (2.0 g scale); BR = linear/mass burn rate (5.0−6.0 g scale); m_a = mass of produced aerosol (2.0 g scale); Y = yield factor; RH = relative humidity during yield determination; m_d = dye content present in produced aerosol; T% = transfer rate; IS = impact sensitivity FS = friction sensitivity; ESD = electrostatic discharge sensitivity; T_{dec} = temperature of decomposition.

References

[1] J. Glück, T. M. Klapötke, M. Rusan and A. P. Shaw, *Propellants, Explosives, Pyrotechnics* **2017**, *42*, 131–141.
[2] J. Glück, T. M. Klapötke and T. Küblböck, *New Journal of Chemistry* **2018**, *42*, 10670–10675.
[3] a) BAM Bundesanstalt für Materialforschung und -prüfung, *Empfehlungen für die Beförderung gefährlicher Güter - Handbuch über Prüfungen und Kriterien*, Bundesanstalt für Materialforschung und -prüfung, Berlin, Germany, **2015**, p; b) Committee of Experts on the Transport of Dangerous Goods, *UN Recommendations on the Transport of Dangerous Goods*, United Nations Economic and Social Council, New York City, NY, USA, **2019**, p.
[4] Subcommittee on Military Smokes and Obscurants, National Research Council, *Toxicity of Military Smokes and Obscurants, Volume 3*, The National Academies Press, Washington, DC, USA, **1999**, p. 108.
[5] T. Küblböck and T. M. Klapötke, *Propellants, Explosives, Pyrotechnics* **2018**, *43*, 1184–1189.
